# The Story of the Insane from Year to Year

**Published:** 1897-01-16

**Authors:** 


### THE STORY OF THE INSANE FROM YEAR TO YEAR.

Ninth Series.

NOTTINGHAM COUNTY ASYLUM.

Here the numbers were almost stationary during 1895, being 332 on January 1st, and 329 on the last day of December. There were 99 admissions, 9 readmissions, 64 recoveries, 6 discharged relieved, and 39 deaths. The percentage of recoveries and admissions stands at 59·25, and that of the deaths on the average number resident at 11·60. Both of these are high; that of the recoveries unusually so. Of the deaths, 16 were from cerebral and spinal diseases, 7 from phthisis, and 4 from pneumonia. Of the admissions, 33 were caused by moral causes, 16 by drink, and 39 by hereditary influence. Dr. Aplin has been making an investigation into alleged increase of insanity in the county of Nottingham, and he arrives at the very consoling conclusion that there is not very much increase; but even his researches show that there is some. In 1885 the ratio of admissions to 10,000 of the population was 3·32, while in 1894 it was 3·76. The visitors say that "Dr. Aplin, and those who are working under his superintendence, do all they can to promote the comfort and recovery of the patients." The weekly rate of maintenance is 9s. 7¾d. per head.

CARMARTHEN COUNTY ASYLUM.

On January 1st the asylum contained 557 patients, and on December 31st the number had increased by only two. There were 71 admissions, 16 readmissions, 27 recoveries, 4 discharged relieved, and 50 deaths. The recoveries stand at 32·1 per cent., and the deaths at 8·99 per cent. Eleven of the deaths were caused by brain diseases, and the same number by consumption. Moral causes account for 16 of the year's admissions, drink for 12, and hereditary influence for 29. In addition to those 12 cases in which drink was the cause of the insanity, Dr. Goodall tells us, what we should like to emphasise, that there is a great mass of cases in which the victim of drink, whilst to all intents incapable of controlling himself and his affairs, is yet not a certifiable lunatic; and there is no proper place for him at present. Nor will there be until the law thinks as much of the misery which the man's family endures as it does now of the sacred liberty of a drunken subject. The border between crime and insanity is well known to be a narrow one in many instances. Four criminals were admitted to the asylum during the year, and in two of them there were strong reasons for believing that insanity existed at the time of, and prior to, the crime; and the other two were probably not responsible for the act. Dr. Goodall thinks there should be a Psychiatric Medical Service in prisons, and if this could be carried out successfully, there can be no doubt that much good would result. Nursing lectures are given, and several of the staff have obtained the certificate of the Medico-Psychological Association. The visitors mark their approbation of the efficient management of the asylum. We learn that the office of medical superintendent at this asylum carries with it no pension. If this be so, we are only too certain that such a system is highly injurious to the best interests of the asylum, and we trust that the visitors and County Council will at once alter the rule and allow the pensions clause of the Lunacy Act to take effect in their asylum. The average weekly cost per head is nearly 7s. 7d.

